# Supplementary figures and images for: The (p)ppGpp-binding GTPase Era promotes rRNA processing and cold adaptation in Staphylococcus aureus
Source: PLoS Genet. 2019 Aug 29;15(8):e1008346. doi: 10.1371/journal.pgen.1008346 (PMC6738653; doi:10.1371/journal.pgen.1008346)

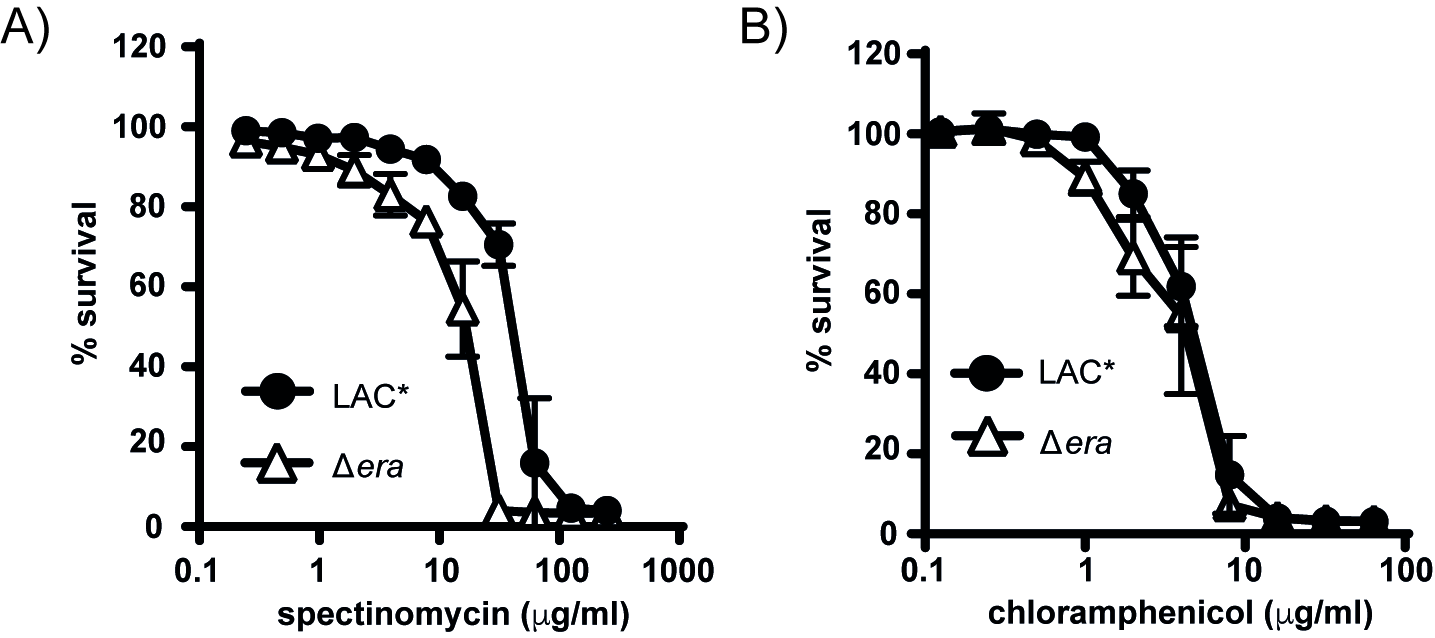

Supplement: S1 Fig — The susceptibility of LAC* and Δera to A) spectinomycin and B) chloramphenicol was measured by growing the strains in 96 well plates with the indicated concentration of each antibiotic. OD600 readings were determined after 24 h of growth and plotted as % survival compared to growth without antibiotic. Experiments were repeated four times and mean and standard deviation plotted. (TIF) [file pgen.1008346.s001.tif]

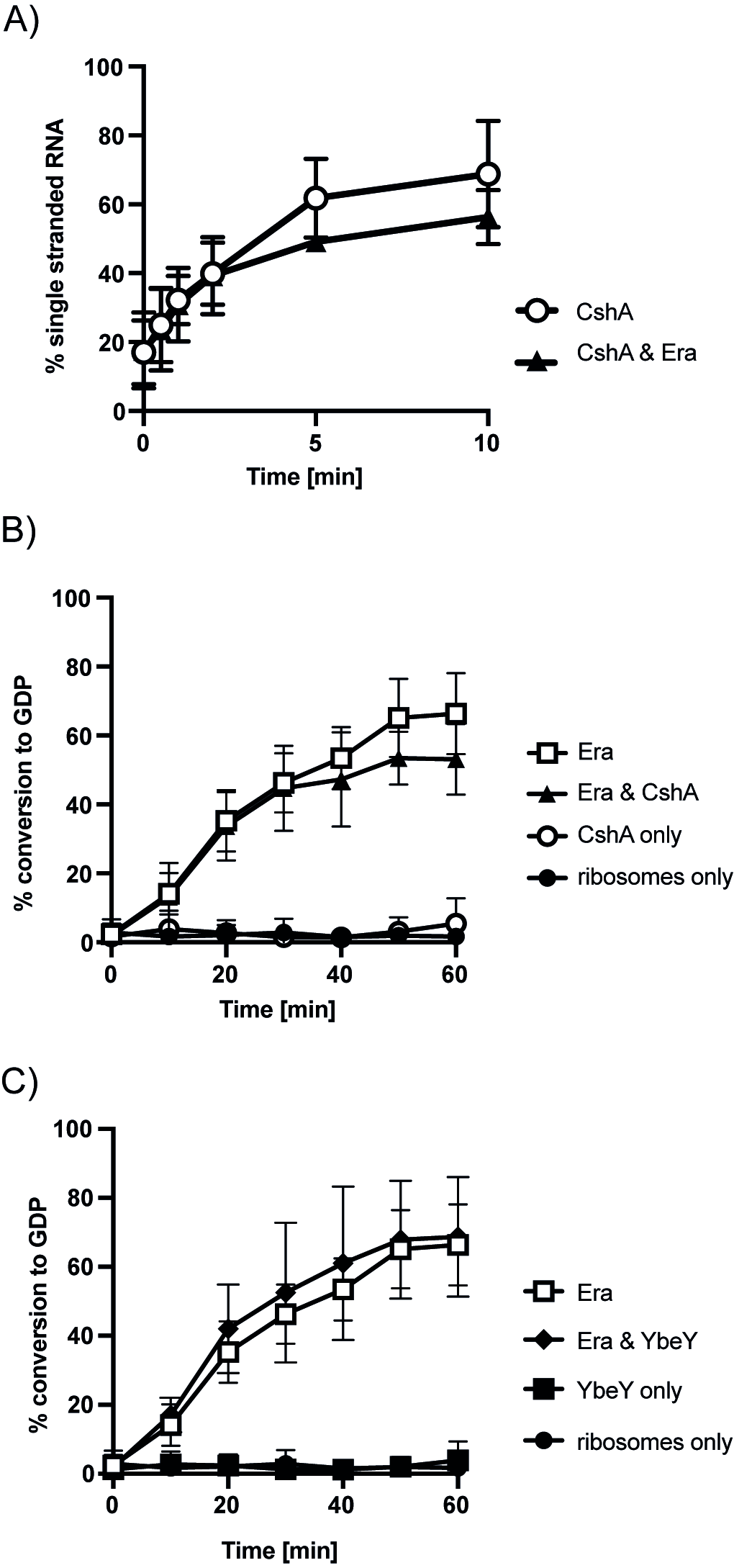

Supplement: S2 Fig — A) The RNA helicase activity of 0.5 μM CshA with and without an equal concentration of Era were determined using a Cy3-labelled double stranded RNA oligomer. Reactions were incubated at 25°C for up to 10 min before analysis on a native page gel. Experiments were repeated three-four times with means and standard deviations shown. Statistical analysis was performed using a two-way ANOVA, followed by Sidak’s multiple comparisons test. B & C) The GTPase activity of 100 nM Era was measured in the presence of an equal amount of ribosomes and 1 μM GTP, plus and minus 100 nM CshA (B) or 100 nM YbeY (C). All reactions contained ribosomes. Reactions lacking Era were included as controls. Hydrolysis of 32P-GTP was monitored by TLC and the percentage GDP formed quantified using ImageJ. Experiments were repeated five times with means and standard deviations shown. Statistical analysis was performed using a two-way ANOVA, followed by Dunnett’s multiple comparisons test. (TIF) [file pgen.1008346.s002.tif]

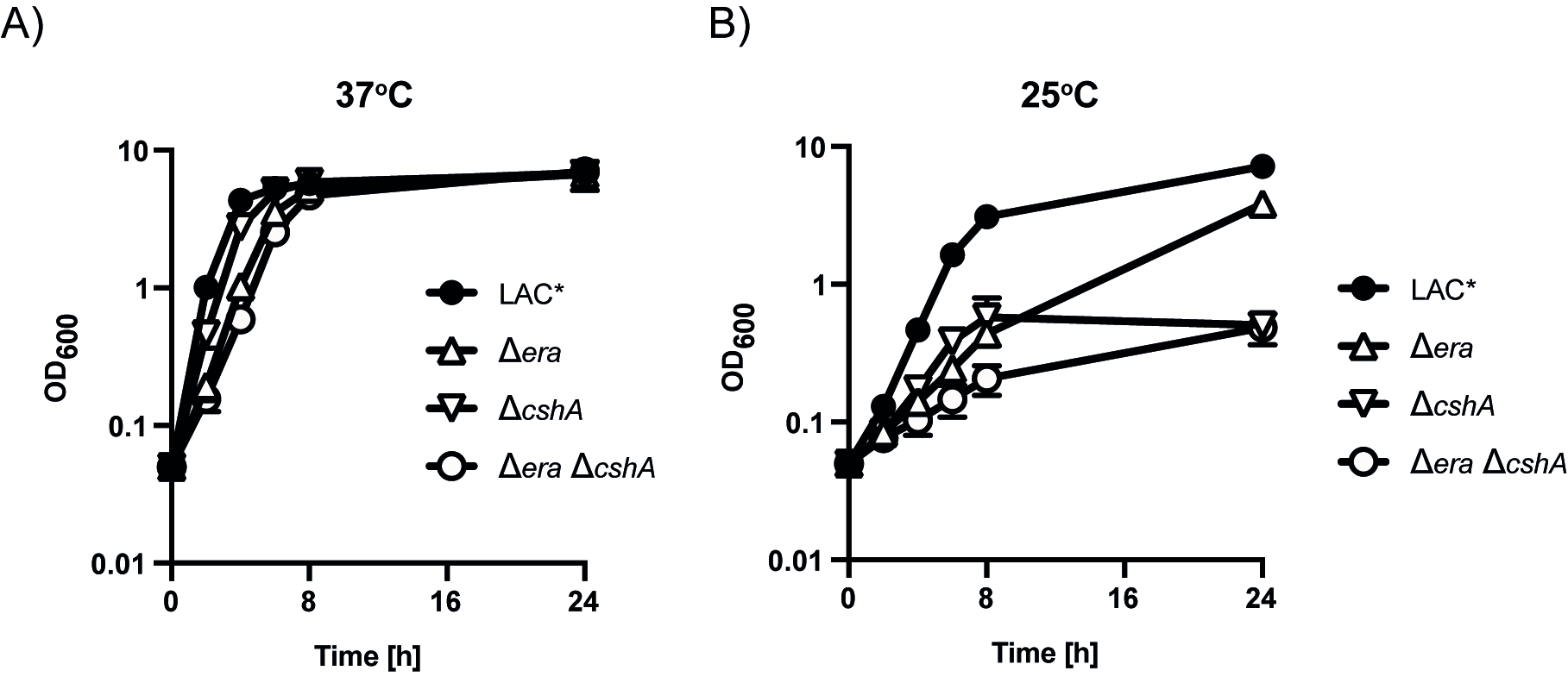

Supplement: S3 Fig — Growth of S. aureus strains LAC*, LAC* Δera, LAC* ΔcshA and LAC* Δera cshA at A) 37°C and B) 25°C. Overnight cultures were diluted to an OD600 of 0.05 and grown for 24 h. Growth curves were performed in triplicate, with averages and standard deviations shown. (TIF) [file pgen.1008346.s003.tif]

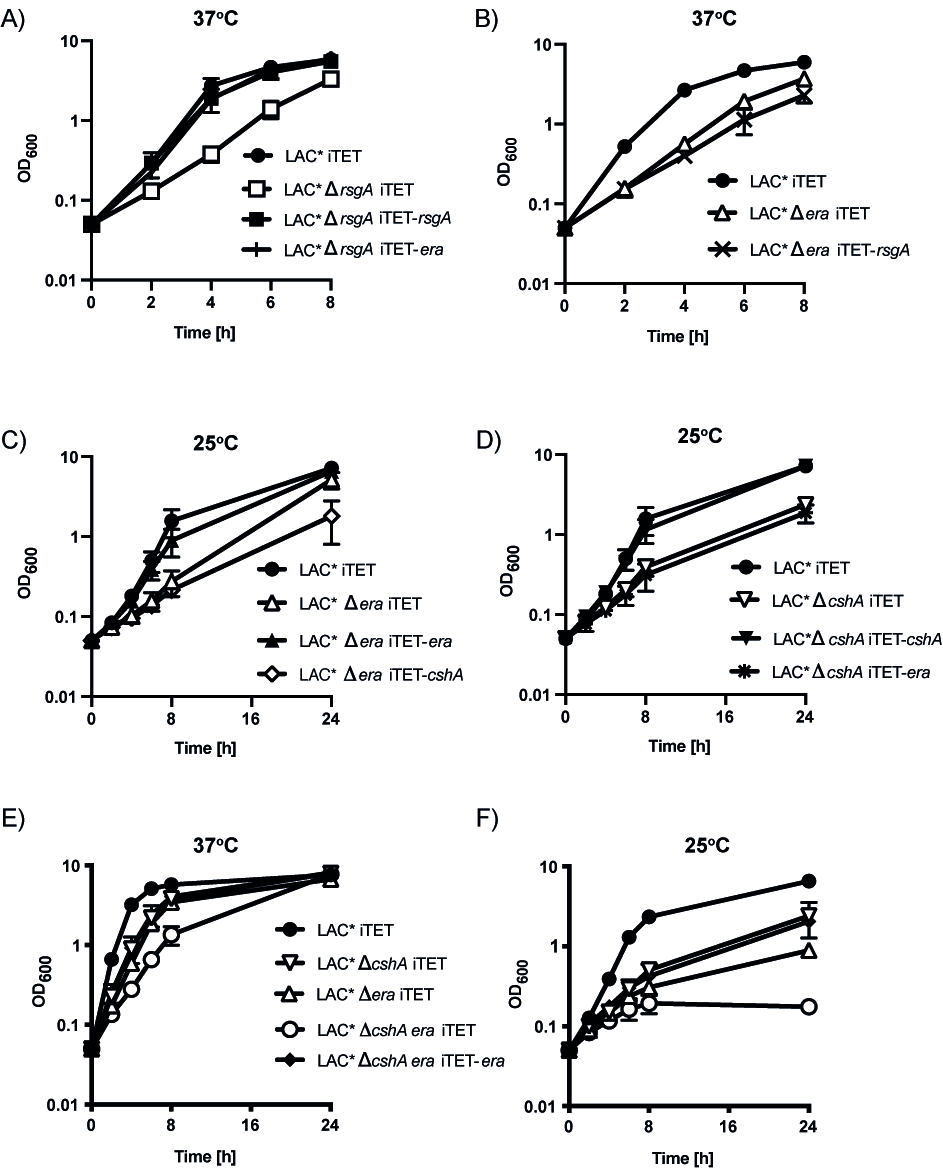

Supplement: S4 Fig — A) Growth of LAC* iTET, LAC* ΔrsgA iTET, LAC* ΔrsgA iTET-rsgA and LAC* ΔrsgA iTET-era at 37°C. B) Growth of LAC* iTET, LAC* Δera iTET and LAC* Δera iTET-rsgA at 37°C. C) Growth of LAC* iTET, LAC* Δera iTET, LAC* Δera iTET-era and LAC* Δera iTET-cshA at 25°C. D) Growth of LAC* iTET, LAC* ΔcshA iTET, LAC* ΔcshA iTET-cshA and LAC* ΔcshA iTET-era at 25°C. E) Growth of LAC* iTET, LAC* ΔcshA iTET, LAC* Δera iTET, LAC* ΔcshA era iTET and LAC* ΔcshA era iTET-era at 37°C. F) Growth of LAC* iTET, LAC* ΔcshA iTET, LAC* Δera iTET, LAC* ΔcshA era iTET and LAC* ΔcshA era iTET-era at 25°C. Overnight cultures were diluted to an OD600 of 0.05 and grown in the presence of 100 ng/ml Atet for 8 h to 24 h at either 37°C or 25°C. Growth curves were performed three to four times, with averages and standard deviations shown. (TIF) [file pgen.1008346.s004.tif]

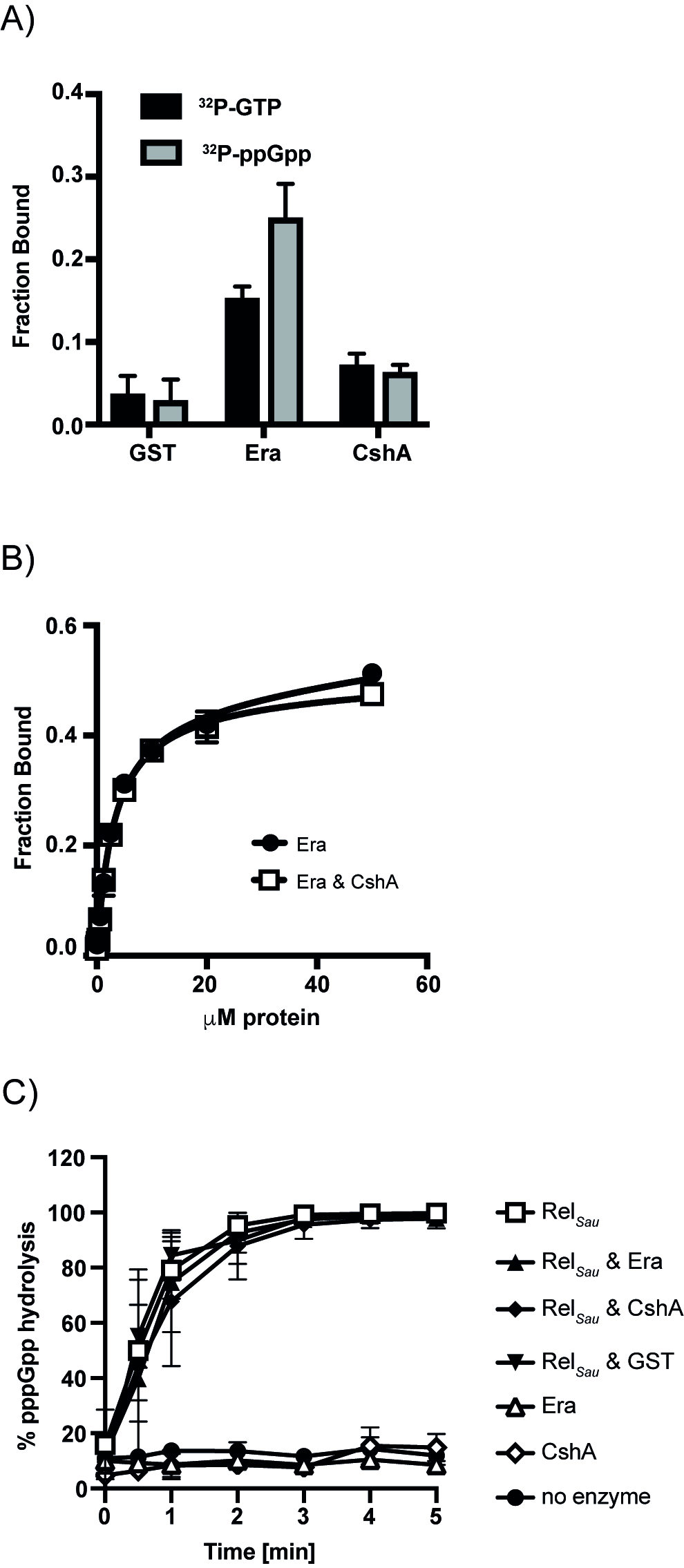

Supplement: S5 Fig — A) DRaCALA binding assays with recombinant GST, GST-CshA and Era-His and 32P-labelled GTP and ppGpp. Quantification was carried out using ImageJ. The average values and standard deviations of triplicate experiments are plotted. B) Binding curves and Kd determination for 32P-ppGpp and Era in the absence and presence of CshA. C) Hydrolysis activity of RelSau on 32P-pppGpp in the absence and presence of Era and CshA. 100 nM of each protein were incubated with 1 μM pppGpp over the course of 5 min at 37°C before reactions were quenched. Reactions lacking RelSau, or including just GST in place of GST-Era/CshA were included as controls. Experiments were repeated two to four times with means and standard deviations plotted. Statistical analysis was performed using a two-way ANOVA, followed by Dunnett’s multiple comparisons test. (TIF) [file pgen.1008346.s005.tif]
