# Supplementary material for: The (p)ppGpp-binding GTPase Era promotes rRNA processing and cold adaptation in Staphylococcus aureus
Source: PLoS Genet. 2019 Aug 29;15(8):e1008346. doi: 10.1371/journal.pgen.1008346 (PMC6738653; doi:10.1371/journal.pgen.1008346)
Supplement: S1 Table — (DOCX) [file pgen.1008346.s006.docx]

**S1 Table. Bacterial strains used in this study**

| **Strain** | **Relevant features** | **Reference** |
| --- | --- | --- |
|  | ***Escherichia coli* strains** |  |
| XL1-Blue | Cloning strain: TetR | Stratagene |
| DH5α | Cloning strain | (Hanahan et al., 1983) |
| BL21 (DE3) | Strain used for protein expression | Novagen |
| BTH101 | BACTH Δ*cya* strain | Euromedex |
| RMC0101 | pTS1 in DH5α | (O’Connell et al., 1993) |
| RMC0109 | pCN55 in XL1-Blue: CarbR | (Charpentier et al., 2004) |
| RMC0114 | pGEX-2TK in DH5α: CarbR | Pharmacia |
| RMC0127 | pKT25 in XL1 Blue: KanR | (Karimova et al., 2001) |
| RMC0128 | pKNT25 in XL1 Blue: KanR | (Karimova et al., 2005) |
| RMC0129 | pUT18 in XL1 Blue: CarbR | (Karimova et al., 2001) |
| RMC0130 | pUT18C in XL1 Blue: CarbR | (Karimova et al., 2001) |
| RMC0138 | pRMC2 in XL1 Blue: CarbR | (Corrigan et al., 2009) |
| RMC0147 | pET28b in XL1-Blue: KanR | Novagen |
| RMC0172 | pVL847-*rel_Sau_* in T7IQ: MBP-His-Rel fusion: GnR, CamR | (Corrigan et al., 2015) |
| RMC1453 | pVL791-*ybeY* in T7IQ: CarbR, CamR | (Corrigan et al., 2015) |
| RMC0397 | pET28b-*era* in XL1-Blue: KanR | This study |
| RMC0482 | pTS1-Δ*era* in XL1-Blue: CarbR | This study |
| RMC0531 | pCN55iTET in XL1-Blue: iTET promoter in pCN55; CarbR | This study |
| RMC0533 | pCN55iTET-*era* in XL1-Blue: CarbR | This study |
| RMC0935 | pCN55iTET-*cshA* in XL1-Blue: CarbR | This study |
| RMC0693 | pKT25-*rel_Sau_* in DH5α: T25 fused to the N-terminus of *rel_Sau_*: KanR | This study |
| RMC0694 | pKNT25-*rel_Sau_* in DH5α: T25 fused to the C-terminus of *rel_Sau_*: KanR | This study |
| RMC0697 | pKT25-*relP* in DH5α: T25 fused to the N-terminus of *relP*: KanR | This study |
| RMC0698 | pKNT25-*relP* in DH5α: T25 fused to the C-terminus of *relP*: KanR | This study |
| RMC0701 | pKT25-*relQ* in DH5α: T25 fused to the N-terminus of *relQ*: KanR | This study |
| RMC0702 | pKNT25-*relQ* in DH5α: T25 fused to the C-terminus of *relQ*: KanR | This study |
| RMC0744 | pKNT25-HD aa41-187 of Rel*_Sau_* in DH5α: KanR | This study |
| RMC0748 | pKNT25-SYN aa247-357 of Rel*_Sau_* in DH5α: KanR | This study |
| RMC0752 | pKNT25-TGS aa402-461 of Rel*_Sau_* in DH5α: KanR | This study |
| RMC0756 | pKNT25-ACT aa657-735 of Rel*_Sau_* in DH5α: KanR | This study |
| RMC0817 | pUT18C-*era* in BTH101: CarbR | This study |
| RMC0821 | pUT18-*era* in BTH101: CarbR | This study |
| RMC0825 | pKNT25-*era* in BTH101: KanR | This study |
| RMC0829 | pKT25-*era* in BTH101: KanR | This study |
| RMC0910 | pUT18C-*cshA* in XL1-Blue: T18 fused to the N-terminus of *cshA*: CarbR | This study |
| RMC0911 | pUT18-*cshA* in XL1-Blue: T18 fused to the C-terminus of *cshA*: CarbR | This study |
| RMC1260 | pUT18C-*ybeZ* in XL1-Blue: T18 fused to the N-terminus of *ybeZ;* CarbR | This study |
| RMC1201 | pUT18C-*ybeY* in XL1-Blue: T18 fused to the N-terminus of *ybeY*: CarbR | This study |
| RMC1202 | pUT18C-*dgkA* in XL1-Blue: T18 fused to the N-terminus of *dgkA*: CarbR | This study |
| RMC1203 | pUT18C-*recO* in XL1-Blue: T18 fused to the N-terminus of *recO*: CarbR | This study |
| RMC1204 | pUT18C-*cdd* in XL1-Blue: T18 fused to the N-terminus of *cdd*: CarbR | This study |
| RMC0909 | pGEX-2TK–*cshA* in XL1-Blue: CarbR | This study |
| RMC1445 | pGEX-2TK–*era* in XL1-Blue: CarbR | This study |
| RMC0945 | pAP118 in DH5α: CamR | [29] |
| RMC0946 | pAF256 in DH5α: plasmid contains HupA fused to SB: CamR | [29] |
| RMC0947 | pAF257 in DH5α: plasmid contains HupA fused to LB: CamR | [29] |
| RMC1098 | pAP118-*era_sb_*-*era_lb_* in XL1-Blue: CamR | This study |
| RMC1099 | pAP118-*era_sb_-cshA_lb_* in XL1-Blue: CamR | This study |
| RMC1101 | pAF257-*era_lb_* in XL1-Blue: CamR | This study |
| RMC1102 | pAF257-*cshA_lb_* in XL1-Blue: CamR | This study |
| RMC1139 | pAF257-*rel_Sau lb_* in XL1-Blue: CamR | This study |
| RMC1156 | pAP118-*era_sb_*-*cshA* 383-506*_lb_* in XL1-Blue: CamR | This study |
| RMC1157 | pAP118-*era_sb_*-*cshA* 1-382*_lb_* in XL1-Blue: CamR | This study |
| RMC1158 | pAF257-*cshA* 383-506*_lb_* in XL1-Blue: CamR | This study |
| RMC1159 | pAF257-*cshA* 1-382*_lb_* in XL1-Blue: CamR | This study |
| RMC1160 | pAP118-*era_sb_*-*ybeZ_lb_* in XL1-Blue: CamR | This study |
| RMC1161 | pAP118-*era_sb_*-*ybeY_lb_* in XL1-Blue: CamR | This study |
| RMC1162 | pAP118-*era_sb_*-*dgkA_lb_* in XL1-Blue: CamR | This study |
| RMC1163 | pAP118-*era_sb_*-*cdd_lb_* in XL1-Blue: CamR | This study |
| RMC1164 | pAP118-*era_sb_*-*recO_lb_* in XL1-Blue: CamR | This study |
| RMC1165 | pAF257-*ybeZ_lb_* in XL1-Blue: CamR | This study |
| RMC1166 | pAF257-*ybeY_lb_* in XL1-Blue: CamR | This study |
| RMC1167 | pAF257-*dgkA_lb_* in XL1-Blue: CamR | This study |
| RMC1168 | pAF257-*cdd_lb_* in XL1-Blue: CamR | This study |
| RMC1169 | pAF257-*recO_lb_* in XL1-Blue: CamR | This study |
| RMC1221 | pAP118-*era* 1-180*_sb_*- *cshA_lb_* in XL1-Blue: CamR | This study |
| RMC1222 | pAF256-*era* 1-180*_sb_* in XL1-Blue: CamR | This study |
| RMC1236 | pAF257-*cshA* 1-221*_lb_* in XL1-Blue: CamR | This study |
| RMC1244 | pAP118-*era_sb_*-*cshA* 1-221*_lb_* in XL1-Blue: CamR | This study |
| RMC1272 | pAF257-*cshA* 222-382*_lb_* in XL1-Blue: CamR | This study |
| RMC1287 | pAP118-*era_sb_*-*cshA* 222-382*_lb_* in XL1-Blue: CamR | This study |
| RMC1444 | pAP118-*cshA_sb_*-*era_lb_* in XL1-Blue: CamR | This study |
| RMC1443 | pAP118-*rel_Sau sb_*-*era_lb_* in XL1-Blue: CamR | This study |
| RMC1454 | pAP118-*era_sb_*-*rel_Sau lb_* in XL1-Blue: CamR | This study |
| RMC1455 | pAP118-*cshA_sb_*-*rel_Sau lb_* in XL1-Blue: CamR | This study |
| RMC1143 | pAP118-*rel_Sau sb_*-*cshA_lb_* in XL1-Blue: CamR | This study |
| RMC1456 | pAF256-*cshA_sb_* in XL1-Blue: CamR | This study |
| RMC1138 | pAF256-*rel_Sau sb_* in XL1-Blue: CamR | This study |
| RMC1259 | pAF256-*era_sb_* in XL1-Blue: CamR | This study |
| RMC1543 | pAP118-*era* 1-180*_sb_*- *rel_Sau_ _lb_* in XL1-Blue: CamR | This study |
|  |  |  |
|  | ***Staphylococcus aureus* strains** |  |
| SEJ1 | RN4220 Δ*spa;* protein A negative derivative of RN4220 | (Gründling et al., 2007) |
| LAC* | Erm sensitive CA-MRSA LAC strain | (Boles et al., 2010) |
| NE565 | USA300 strain JE2 with transposon insertion in *cshA*: ErmR | [23] |
| RMC0358 | LAC* Δ*rsgA*: ErmR | [22] |
| RMC0447 | RN4220 pTET: TetR | (Boles et al., 2010) |
| RMC0552 | RN4220Δ*spa* Δ*era* pCN55iTET-*era*: TetR, SpecR | This study |
| RMC0562 | LAC* pCN55iTET: SpecR | This study |
| RMC0650 | LAC* Δ*era*: TetR | This study |
| RMC0813 | LAC* Δ*era* pCN55iTET: TetR, SpecR | This study |
| RMC0814 | LAC* Δ*era* pCN55iTET-*era*: TetR, SpecR | This study |
| RMC0908 | LAC* Δ*cshA*: ErmR | This study |
| RMC1030 | LAC* Δ*cshA* pCN55iTET: ErmR, SpecR | This study |
| RMC1031 | LAC* Δ*cshA* pCN55iTET-*era*: ErmR, SpecR | This study |
| RMC1032 | LAC* Δ*cshA* pCN55iTET-*cshA*: ErmR, SpecR | This study |
| RMC1033 | LAC* Δ*cshA* Δ*era*: ErmR TetR | This study |
| RMC1034 | LAC* Δ*era* pCN55iTET-*cshA*: TetR, SpecR | This study |
| RMC0890 | LAC* Δ*rsgA* pCN55iTET: ErmR, SpecR | This study |
| RMC0891 | LAC* Δ*rsgA* pCN55iTET-*era*: ErmR, SpecR | This study |
| RMC1565 | LAC* Δ*cshA* Δ*era* pCN55iTET-*era*: ErmR TetR, SpecR | This study |
| RMC1562 | LAC* Δ*rsgA* pCN55iTET-*rsgA*: ErmR, SpecR | This study |
| RMC1563 | LAC* Δ*era* pCN55iTET-*rsgA*: TetR, SpecR | This study |
|  |  |  |

Antibiotics were used at the following concentrations - for *E. coli* cultures: carbenicillin (CarbR) 50-150 μg/ml; kanamycin (KanR) 30 μg/ml; chloramphenicol (CamR) 20 μg/ml, gentamicin (GnR) 20 μg/ml. For *S. aureus* cultures: erythromycin (ErmR) 10 μg/ml; chloramphenicol (CamR) 7.5 to 10 μg/ml; spectinomycin (SpecR) 250 μg/ml; tetracycline (TetR) 2 μg/ml. IPTG was used at 1 mM and anhydrotetracycline (Atet) at 100 ng/ml.

Hanahan D. Studies on transformation of *Escherichia coli* with plasmids. J Mol Biol. 1983;166:557-580.

O'Connell C, Pattee PA, Foster TJ. Sequence and mapping of the *aroA* gene of *Staphylococcus aureus* 8325-4. J Gen Microbiol. 1993;139:1449-1460.

Charpentier E, Anton AI, Barry P, Alfonso B, Fang Y, Novick RP. Novel cassette-based shuttle vector system for gram-positive bacteria. Appl Environ Microbiol. 2004;70:6076-6085.

Karimova G, Ullmann A, Ladant D. Protein-protein interaction between *Bacillus stearothermophilus* tyrosyl-tRNA synthetase subdomains revealed by a bacterial two-hybrid system. J Mol Microbiol Biotechnol. 2001;3:73-82.

Karimova G, Dautin N, Ladant D. Interaction network among *Escherichia coli* membrane proteins involved in cell division as revealed by bacterial two-hybrid analysis. J Bacteriol. 2005;187:2233-2243.

Corrigan RM, Foster TJ. An improved tetracycline-inducible expression vector for *Staphylococcus aureus*. Plasmid. 2009;61:126-129.

Corrigan RM, Bowman L, Willis AR, Kaever V, Grundling A. Cross-talk between two nucleotide-signaling pathways in *Staphylococcus aureus*. J Biol Chem. 2015;290:5826-5839.

Gründling A, Schneewind O. Genes Required for Glycolipid Synthesis and Lipoteichoic Acid Anchoring in *Staphylococcus aureus*. J Bacteriol. 2007;189:2521-2530.

Boles BR, Thoendel M, Roth AJ, Horswill AR. Identification of genes involved in polysaccharide-independent *Staphylococcus aureus* biofilm formation. PLoS One. 2010;5:e10146.
