# Supplementary material for: The (p)ppGpp-binding GTPase Era promotes rRNA processing and cold adaptation in Staphylococcus aureus
Source: PLoS Genet. 2019 Aug 29;15(8):e1008346. doi: 10.1371/journal.pgen.1008346 (PMC6738653; doi:10.1371/journal.pgen.1008346)
Supplement: S2 Table — (DOCX) [file pgen.1008346.s007.docx]

**S2 Table. Primers used in this study**

| **Number** | **Name** | **Sequence** |
| --- | --- | --- |
| RMC068 | F-NdeI-Era | cccCATATGACAGAACATAAATCAGCATTTGT |
| RMC069 | R-BamHI-Era | cccGGATCCTTAATCTTGGTCTTCAACATAACC |
| RMC178 | F-BamHI-Era | aaaGGATCCgaagaagatgagccagagattg |
| RMC179 | R-BamHI-Era | AAAGGATCCCACCTTATTGTGTAACATTCGAC |
| RMC151 | R-up FtetAM | aataattttcatttattctaaatcctttcctgaaaa |
| RMC152 | F-tetAM | GATTTAGAATAAATGAAAATTATTAATATTGGAGTT |
| RMC153 | R-tetAM | ccacttttaagactaagttattttattgaacatata |
| RMC154 | F-down Rtet | AAAATAACTTAGTCTTAAAAGTGGTGAAGATAATTG |
| RMC157 | F-KpnI-Era | ggggGGTACCtcaggaaaggatttagaataaatg |
| RMC158 | R-SacI-Era | CCCCGAGCTCTTAATCTTGGTCTTCAACATAACC |
| RMC185 | R-NarI-tetR | CCTTGGCGCCTTAAGACCCACTTTCACATTTAAGTTG |
| RMC186 | F-XmaI-pRMC2 | TCCCCCGGGCGGAATTCGAGCTCAGATCTGTTAACGGTACCATC |
| RMC258 | F-XbaI-Rel*_Sau_* | ggggTCTAGAgaacaacgaatatcc |
| RMC259 | R-KpnI- Rel*_Sau_* | GGGGGTACCTTCCAAACTCTTGTTAC |
| RMC260 | F-XbaI-RelP | gggTCTAGAgtatgtagatcgaaaacc |
| RMC261 | R-KpnI-RelP | GGGGGTACCTCTGTTATTTCAGAATG |
| RMC262 | F-XbaI-RelQ | gggTCTAGAgaatcaatgggatcag |
| RMC263 | R-KpnI-RelQ | GGGGGTACCTCATTTTCATGTTTTTTAGAACG |
| RMC266 | F-XbaI-HD | gggTCTAGAtattgcttatgaagcac |
| RMC267 | R-KpnI-HD | GGGGGTACCTTAATACCAAGACGATGTG |
| RMC268 | F-XbaI-Synth | gggTCTAGAtggtagacctaaacatatttac |
| RMC269 | R-KpnI-Synth | GGGGGTACCTTTTTACCTTCTTTGTAAGC |
| RMC270 | F-XbaI-TGS | gggTCTAGAagtatacgcatttacccc |
| RMC271 | R-KpnI-TGS | GGGGGTACCCTAGTACGTATTTCAAC |
| RMC272 | F-XbaI-ACT | gggTCTAGAtcaaaaatatcaggttg |
| RMC326 | R-KpnI-Era | CCCGGTACCCCATCTTGGTCTTCAACATAACCAATTTG |
| RMC330 | F-XbaI-Era | gggTCTAGAGACAGAACATAAATCAGGATTTGTTTC |
| RMC401 | F-SmaI-CshA | AGTCCCCGGGGCAAAATTTTAAAGAACTAG |
| RMC402 | R-SmaI-CshA | agctCCCGGGtttttgatggtcagcaaatgtg |
| RMC407 | F-XbaI-CshA | GGGTCTAGATTGCAAAATTTTAAAGAACTAGGGATTTC |
| RMC408 | R-KpnI-CshA | cccGGTACCccTTTTTGATGGTCAGCAAATGTGc |
| RMC434 | F-XbaI-YbeZ | GGGTCTAGAGATGAAAAGGAGCGCGTG |
| RMC435 | R-KpnI-YbeZ | cccGGTACCggattctctccttcataatgttcaatgatc |
| RMC436 | F-XbaI-YbeY | GGGTCTAGAGATGTTTACGATAGATTTTAGCGATC |
| RMC437 | R-KpnI-YbeY | cccGGTACCgggtctcgtgttaatccatatgcg |
| RMC438 | F-XbaI-DgkA | GGGTCTAGAGATGAAAAGGTTTAAATATGCACTTG |
| RMC439 | R-KpnI-DgkA | cccGGTACCggaaataacgctataaaatgtggtaaaaatac |
| RMC440 | F-XbaI-RecO | GGGTCTAGAGATGCGCCAAAAAGGGATTATC |
| RMC441 | R-KpnI-RecO | cccGGTACCggttgttccaatctttttaattggttg |
| RMC442 | F-XbaI-Cdd | GGGTCTAGAGATGAGTTATCAACCTCATTATTTTCAAG |
| RMC443 | R-KpnI-Cdd | cccgaatccGGttctaaatcctttcctgaaaatcc |
| RMC447 | F-KpnI-CshA | ggggGGTACCcaggtaaaaaggagaattattttg |
| RMC448 | R-SacI-CshA | CCCCGAGCTCTTATTTTTGATGGTCAGCAAATGT |
| RMC483 | F-SacI-Era | gggGAGCTCtttcaggaaaggatttagaataaa |
| RMC484 | R-XhoI-Era | gggCTCGAGaatcttggtcttcaacataaccaa |
| RMC485 | F-PvuI-Era | GGGCGATCGTTTCAGGAAAGGATTTAGAATAAA |
| RMC486 | R-NotI-Era | gggGCGGCCGCatcttggtcttcaacataaccaat |
| RMC487 | F-PvuI-CshA | GGGCGATCGCAGGTAAAAAGGAGAATTATTTTG |
| RMC488 | R-NotI-CshA | gggGCGGCCGCtttttgatggtcagcaaatgtgc |
| RMC497 | R-NotI-CshA382 | gggGCGGCCGCgatgtcatcttcacgtgcttg |
| RMC498 | F-PvuI-CshA383 | gggCGATCGaaaaaggagaattattaaagaaaaagttgaaaactggatg |
| RMC499 | F-PvuI-YbeZ | gggCGATCGgcatcatagaatgaatataaatgatat |
| RMC500 | R-NotI-YbeZ | gggGCGGCCGCattctctccttcataatgttcaatg |
| RMC501 | F-PvuI-YbeY | gggCGATCGgaacattatgaaggagagaattaa |
| RMC502 | R-NotI-YbeY | gggGCGGCCGCgtctcgtgttaatccatatgc |
| RMC503 | F-PvuI-DgkA | gggCGATCGcatatggattaacacgagactaatt |
| RMC504 | R-NotI-DgkA | gggGCGGCCGCaaataacgctataaaatgtgg |
| RMC505 | F-PvuI-Cdd | gggCGATCGcgttattttagggaggcatat |
| RMC506 | R-NotI-Cdd | gggGCGGCCGCttctaaatcctttcctgaaaatcc |
| RMC507 | F-PvuI-RecO | gggCGATCGcttaaaagtggtgaagataattgtta |
| RMC508 | R-NotI-RecO | gggGCGGCCGCttgttccaatctttttaattggttg |
| RMC516 | R-XhoI-Era 1-180 | gggCTCGAGatcatctggataatatttaggtcc |
| RMC517 | R-NotI-CshA221 | gggGCGGCCGCaattgtatagaattcttcgatttg |
| RMC533 | F-PvuI-CshA222 | gggCGATCGaaaaaggagaattattgttaaagaattagagaaatttgatac |
| RMC570 | 5'-Leader-F | ttagtatttatgagctaatcaaacatc |
| RMC571 | 5'-Leader-R | aaaatattatccggtattagctcc |
| RMC379 | Internal-F | AGCTTAGTTGCCATCATTAAGTTGG |
| RMC380 | Internal-R | GTTGcAGACTACAATCCGAACTG |
| RMC572 | 3'trailer-F | catgctacggtgaatacgtt |
| RMC548 | 3'trailer-R | acgttattccgcatcttctg |
| RMC573 | Rho-F | GAAGCTGCTGAAGTCG |
| RMC574 | Rho-R | GAATGCTTTTGGTTTGTGTAa |
| RMC657 | F-BamHI-Era | gggGGATCCatgacagaacataaatcaggatttg |
| RMC658 | R-EcoRI-Era | gggGAATTCttaatcttggtcttcaacataacc |
| RMC659 | R-XhoI-CshA | gggCTCGAGAtttttgatggtcagcaaatgtgc |
|  |  |  |

Restriction sites in primer sequences are underlined
