# Supplementary material for: The (p)ppGpp-binding GTPase Era promotes rRNA processing and cold adaptation in Staphylococcus aureus
Source: PLoS Genet. 2019 Aug 29;15(8):e1008346. doi: 10.1371/journal.pgen.1008346 (PMC6738653; doi:10.1371/journal.pgen.1008346)
Supplement: S1 Protocol — (DOCX) [file pgen.1008346.s008.docx]

**Plasmid and strain construction.** Strains used in this study are listed in S1 Table and primers are listed in S2 Table. Strain construction is outlined below:

*S. aureus complementation plasmids*: Plasmid pCN55iTET was constructed by amplifying the iTET promoter from pRMC2 with primers RMC185/186 and cloning into the NarI/XmaI sites of pCN55. pCN55iTET-*era* and pCN55iTET-*cshA* were constructed by amplifying the *era* and *cshA* genes with primers RMC157/158 and RMC447/448 respectively, from LAC* genomic DNA and cloning into the KpnI/SacI sites of pCN55iTET.

*S. aureus gene deletion strains*: For the deletion of the *era* gene, 1 kb fragments up- and downstream of *era* were amplified from LAC* genomic DNA using primer pairs RMC178/151 and RMC154/179, which incorporate 5' and 3' BamHI sites. A *tetAM* gene was amplified from plasmid pTET using primer pair RMC152/153. Purified PCR products were then fused by SOE PCR using primers RMC178/179, digested with BamHI and cloned into the allelic exchange vector pTS1 yielding plasmid pTS1-Δ*era*. This plasmid was then electroporated into SEJ1 and stably maintained at 30°C in the presence of 10 μg/ml Cam. Shifting the temperature to 43°C resulted in insertion of the plasmid into the chromosome. The covering plasmid pCN55iTET-*era* was introduced into the strain, as it was at this point unknown if this gene was essential for growth or not. Downshift of the temperature to 30°C in the absence of chloramphenicol but in the presence of spectinomycin and Atet to select for and induce expression of Era from the plasmid, resulted in excision of the pTS1 plasmid and replaced the chromosomal copy of the *era* gene with the *tetAM* gene. The tetracycline marked *era* deletion was then transduced into a fresh LAC* strain background yielding LAC*Δ*era*.

*S. aureus luciferase strains*: plasmids pAP118-*era_sb_*-*era_lb_*, pAP118-*era_sb_*-*cshA_lb_,* pAP118-*era_sb_*-*rel_Saulb_,* pAP118-*rel_Sausb_*-*era_lb_,* pAP118-*rel_Sausb_*-*cshA_lb_,* pAP118-*cshA_sb_*-*era_lb_,* pAP118-*cshA_sb_*-*rel_Saulb_,* pAP118-*era_sb_*-*cshA* 1-221_lb_, pAP118-*era_sb_*-*cshA* 1-382_lb_, pAP118-*era_sb_*-*cshA* 222-382_lb_, pAP118-*era_sb_*-*cshA* 383-506_lb_, pAP118-*era_sb_*-*ybeZ_lb_,* pAP118-*era_sb_*-*ybeY_lb_,* pAP118-*era_sb_*-*dgkA_lb_,* pAP118-*era_sb_*-*cdd_lb_*, pAP118-*era_sb_*-*recO_lb_,* and pAP118-*era* 1-180*_sb_*-*cshA_lb_* were constructed by first amplifying either the full-length *era, rel_Sau_* or *cshA* genes, or the *era* 1-180 amino acid fragment, with the primers listed in Table S2 and cloning into the SacI/XhoI sites of pAP118, fusing each gene to the small bit (*sb*) of the nanoluc luciferase gene. pAP118 vectors were subsequently digested with PvuI and NotI. The appropriate genes for fusing to the large bit (*lb*) of the nanoluc were amplified with primers as specified in Table S2 and cloned into digested pAP118 vectors*.* The control strains pAF256-*era_sb_*, pAF256-*rel_Sausb_,* pAF256-*cshA_sb,_* pAF256-*era* 1-180*_sb_* and pAF257-*cshA_lb_* were created by amplifying the respective genes and cloning into pAF256 or pAF257 digested with SacI/XhoI or PvuI/NotI to first drop out the native HupA. All plasmids were electroporated into RN4220 Δ*spa,* before isolation and electroporation into the appropriate background.

*E. coli strains and plasmids:* All pKT25, pKNT25, pUT18 and pUT18C plasmids were constructed by amplifying the appropriate gene from LAC* genomic DNA with the primers listed in Table S2. PCR products were digested with XbaI/KpnI and cloned into digested vector. Plasmid pET28b-*era* for expression of His-Era was constructed by amplifying *era* with primers RMC68/69 and cloning into the BamHI and NdeI sites of digested pET28b. The GST-CshA and GST-Era producing clones pGEX-2TK-*cshA* and pGEX-2TK-*era* were created by cloning full-length *cshA* and *era* with the primers RMC401/402 and RMC657/658 into the SmaI site for *cshA* and BamHI/EcoRI sites for *era* of pGEX-2TK, respectively. All plasmids were initially transformed into *E. coli* strain XL1-Blue and sequences of all inserts were verified by fluorescence automated sequencing by GATC. For protein expression and purification, all pET28b and pGEX-2TK derived plasmids were transformed into *E. coli* strain BL21 (DE3).
